# Troponin-T as predictor of mortality in patients attending the emergency department with atrial fibrillation

**DOI:** 10.1186/s12872-024-04388-8

**Published:** 2024-12-20

**Authors:** Serkan Celik, Linus Bodeström Eriksson, Jakob Hytting, Annette Waldemar, Panagiotis Mallios, Amanda Berggren, Ellen Oscarsson, Christofer Digerfeldt, Magnus Wijkman, Laila Hubbert

**Affiliations:** 1 Department of Cardiology and Department of Health, Medicine, and Caring Sciences, Linkoping University, Norrkoping, SE-601 82 Sweden; 2 Department of Medicine and Department of Health, Medicine, and Caring Sciences, Linkoping University, Norrkoping, Sweden

**Keywords:** Atrial fibrillation, Emergency department, Troponin-T, Mortality

## Abstract

**Background:**

High-sensitive Troponin-T (hsTnT) is often increased in acute illness and may be of prognostic importance in patients with atrial fibrillation (AF). The aim of this study was to analyse the characteristics and data of patients attending the emergency department (ED) with AF to determine whether age-adjusted hsTnT levels can predict mortality.

**Methods:**

This retrospective, single centre, register-based cohort study included all patients ≥ 18 years attending the emergency department during 2018 and 2020 with a primary diagnosis at the ED of AF and sampled for hsTnT. Symptoms, comorbidities, lab results, and characteristics were registered. Patients were divided into groups based on hsTnT level (< 15, 15–50, and > 50 ng/L). Primary outcomes: 30-day and 1-year mortality.

**Results:**

A total of 625 patients were included (median age 72, and 45% female). All-cause mortality was 2% at 30 days and 8% at 1-year. The hazard ratio (HR) for 30-day mortality was 4.17 (95% confidence interval (CI) 0.49–35.79, *p* = 0.192) for hsTnT 15–50 ng/L and 9.64 (95% CI 0.98–95.30, *p* = 0.053) for hsTnT > 50 ng/L compared to hsTnT < 15 ng/L when adjusted for age. The HR for 1-year mortality was 4.82 (95% CI 1.81–12.82, *p* = 0.002) for hsTnT 15–50 ng/L and 9.70 (95% CI 3.27–28.74, *p* < 0.001) for hsTnT > 50 ng/L compared to hsTnT < 15 ng/L when adjusted for age.

**Conclusions:**

Elevated hsTnT levels increase the risk for 30-day and 1-year mortality independently of age. Both mild and major elevation of hsTnT levels is associated with increased risk for 1-year mortality regardless of age.

**Supplementary Information:**

The online version contains supplementary material available at 10.1186/s12872-024-04388-8.

## Introduction

Atrial fibrillation/flutter (AF) is the most prevalent cardiac arrhythmia worldwide [1]. The prevalence of AF in Sweden is estimated to 3% in the adult population and increases to more than 10% in those 80 years or older [2]. Approximately 1–2% of all visits to the emergency department (ED) and 1.5-2% of all hospital admissions are due to AF, and as life expectancy increases, this figure is expected to double by 2050 [3]. Even though > 50% with paroxysmal AF spontaneously revert to sinus rhythm within 8–16 h after onset, 30% of patients with AF seek the ED for medical care [4, 5].

High-sensitivity troponin-T (hsTnT) is often increased in acute coronary syndrome, coronary artery disease (CAD), and acute illness [6]. Levels of hsTnT may also be of prognostic value in patients with AF and it seems to be associated with cardiovascular complications and mortality [7, 8]. However, there is conflicting evidence whether mildly elevated hsTnT levels are associated with adverse outcomes [9–11]. Furthermore, there are, to the best of our knowledge, few studies from the emergency department (ED) on the use of mildy elevated hsTnT as a predictor of adverse outcomes in AF patients adjusted for age. The aim of this study was to analyse the association of different hsTnT levels on 30-day and 1-year mortality in patients attending the emergency department (ED) with AF.

## Methods

### Study cohort

All patients ≥ 18 years who attended the ED during the years 2018 and 2020 with the primary diagnosis AF (ICD-10 I48) at Vrinnevi Hospital in Norrkoping, Sweden (catchment area population 170,000), and who were sampled for hsTnT were eligible. Patients were divided into hsTnT level groups: <15 ng/L, 15–50 ng/L, and > 50 ng/L.

### Data sources

This was a retrospective, single centre, register-based cohort study where data were retrieved from REBUS (Region Ostergotland decision support and follow-up system) register and medical records. The following were registered: date attending the ED, diagnosis, age and sex, length of stay on the ED, date of discharge, hospital admission or not, symptoms (palpitations, dyspnoea, fatigue, chest pain, dizziness, and syncope), duration of AF, and duration of symptoms. Baseline risk factors at the time of diagnosis such as body mass index (BMI) > 30 kg/m^2^, Type-2 diabetes mellitus (T2DM), smoking, and blood pressure were taken from medical records as well as any of the comorbidities heart failure (HF) ICD-10 I50, coronary artery disease (CAD) ICD-10 I20-25, and hypertension (HT) ICD-10 I10. Electrocardiography (ECG) for heart rhythm and heart rate, blood sampling for hsTnT, haemoglobin, C-reactive protein (CRP), and Creatinine were performed according to routine hospital care with the patients not fasting. Multiple hsTnT sampling was defined as two or more samples taken.

In the ED the Rapid Emergency Triage and Treatment System (RETTS) is used in all admitted patients. This triage system uses the main symptom of complaint for the determination of triage priority and suggests certain blood sampling, such as hsTnT in patients with e.g. chest discomfort [12]. Physicians use this system together with their clinical evaluation of the patient to decide the need for specific blood sampling and other adequate examinations such as ECG and imaging.

Date of death was collected from the medical records more than one year after the initial visit.

Data from the register and medical records were merged for each patient using the unique national identification number assigned to every Swedish resident at birth or when granted permanent residency.

### Outcome measures

The primary outcome measures were all-cause mortality at 30 days and 1 year. Age-adjusted hsTnT was analysed as a predictor for all-cause mortality and patients were censored at the time of death, 30 days, and 1 year.

### Statistical analysis

Kolmogorov-Smirnov tests were used on continuous variables to determine whether their distributions were normal or skewed. Continuous data with skewed distribution were presented as median and interquartile range (IQR: Q1-Q3). As the assay used for assessment of CRP could not measure < 5 mg/L, such values were set to 3 mg/L. For categorical variables, between-group differences were tested for statistical significance using Chi-square test.

For continuous variables, the Mann-Whitney U-test was used on non-normally distributed data when comparing two groups of independent samples, and the Kruskal-Wallis test was used when comparing three or more groups. To examine possible associations betweenhsTnT and mortality, participants were divided into three hsTnT groups (< 15, 15–50 and > 50 ng/L) and comparisons were made between the groups. Spearman’s correlation was used to quantify correlations between hsTnT level and age.

Hazard ratios (HR) with 95% confidence interval (CI) were calculated using the Cox proportional hazards model and was used to examine possible associations between hsTnT groups and mortality. Kaplan-Meier curves were used to visualise 1-year cumulative mortality for hsTnT groups and for between-group comparisons, the Log rank test was used. The significance level was set at a p-value of < 0.05. IBM SPSS, Statistics, 28.0 (Armonk, NY, USA) was used for statistical analyses.

## Results

**Fig. 1:**
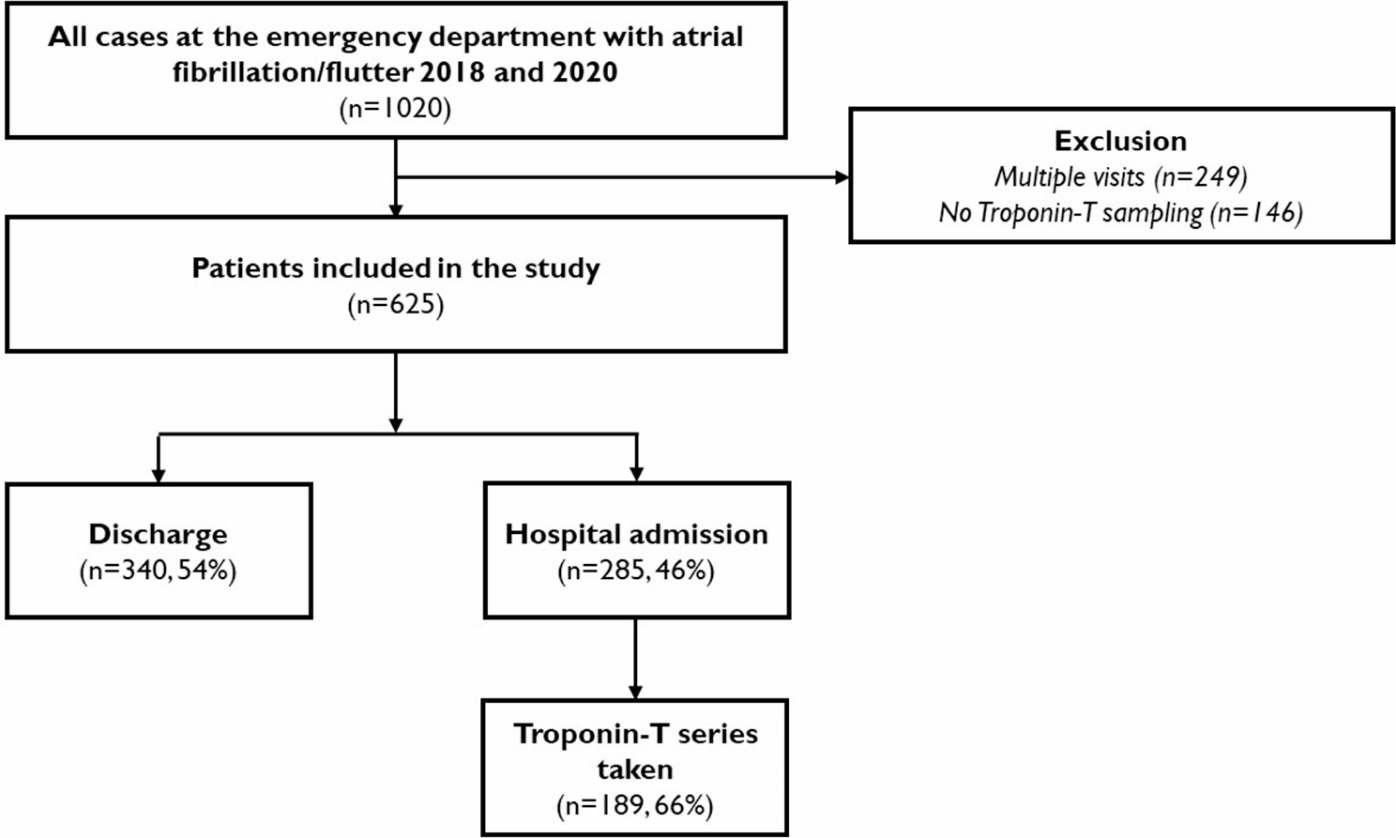
Flow diagram of patients at the emergency department. The relative (%) multiple sampling taken was calculated from the number of patients admitted

A total of 1020 patients attending the emergency department with AF during 2018 and 2020 were identified. Of these, 249 cases were excluded due to multiple visits and 146 because of the absence of hsTnT sampling (unknown reasons), leaving 625 patients included in the study (Fig. 1). 163 patients (26%) had HF, 107 patients (17%) had CAD, and 440 patients (70%) had HT (> 140/80mmHg). 15% were smokers (91 patients), 19% (116 patients) had T2DM, and 28% (174 patients) had a BMI > 30 kg/m^2^. Median creatinine level was 83 (71–100) µmol/L (Table 1).

**Table 1:** Demographic and clinical characteristics of 625 patients with atrial fibrillation/flutter (AF) at the emergency department (ED) sampled for high-sensitive Troponin-T (hsTnT), Vrinnevi Hospital, Norrkoping, Sweden, during years 2018 and 2020

hsTnT (ng/L) *n* (%)	< 15 316 (51)	15–50 259 (41)	> 50 50 (8)	All patients 625 (100)	*p*-value
**Median age**, years (Q1-Q3)	68 (58–74)	77 (70–81)	80 (69–87)	72 (63–79)	< 0.001
**Female**, n (%)	147 (47)	115 (44)	22 (44)	284 (45)	0.859
Duration of AF, months (Q1-Q3)	10 (0–68)	0 (0–46)	0 (0–23)	0.4 (0–57)	0.006
ECG with AF, n (%)	235 (74)	191 (74)	40 (80)	466 (75)	0.657
ECG with AFl, n (%)	39 (12)	41 (16)	7 (14)	87 (14)	0.480
ECG with SR, n (%)	36 (11)	18 (7)	3 (6)	57 (9)	0.140
Heart rate, bpm (Q1-Q3)	126 (99–141)	124 (102–141)	137 (111–146)	126 (100–141)	0.058
**Symptom**					
Duration of symptoms, days median (Q1-Q3)	1 (1–2)	1 (1–4)	2 (1–5)	1 (1–3)	0.004
Palpitations, n (%)	225 (71)	120 (46)	15 (30)	360 (58)	< 0.001
Dyspnoea, n (%)	96 (30)	112 (43)	22 (44)	230 (37)	0.004
Chest pain, n (%)	56 (18)	70 (27)	9 (18)	135 (22)	0.027
Fatigue, n (%)	71 (22)	51 (20)	21 (42)	143 (23)	0.002
Dizziness, n (%)	68 (22)	55 (21)	9 (18)	132 (21)	0.849
Syncope, n (%)	11 (3)	7 (3)	2 (4)	20 (3)	0.827
**Comorbidity and CVRF**					
Smoker, n (%)	49 (16)	32 (12)	10 (20)	91 (15)	0.312
Heart failure, n (%)	82 (26)	69 (27)	12 (24)	163 (26)	0.924
Coronary artery disease, n (%)	52 (16)	43 (17)	12 (24)	107 (17)	0.403
Type-2 diabetes, n (%)	58 (18)	50 (19)	8 (16)	116 (19)	0.852
Hypertension, n (%)	230 (73)	181 (70)	29 (58)	440 (70)	0.101
Systolic BP, mmHg (Q1-Q3)	140 (125–152)	135 (120–150)	130 (114–142)	136 (121–150)	0.090
Diastolic BP, mmHg (Q1-Q3)	90 (79–100)	87 (77–100)	81 (76–99)	88 (78–100)	0.149
BMI > 30 kg/m^2^, n (%)	91 (29)	70 (27)	13 (26)	174 (28)	0.290
**Lab results**					
Haemoglobin, g/L (Q1-Q3)	148 (139–157)	140 (129–148)	131 (120–151)	144 (133–153)	< 0.001
CRP, mg/L (Q1-Q3)	3 (3–3)	3 (3–10)	13 (3–84)	3 (3–7)	< 0.001
Creatinine, µmol/L (Q1-Q3)	78 (67–90)	88 (74–108)	108 (84–146)	83 (69–96)	< 0.001

Data are presented as number (%) or median and quartile range (Q1-Q3) if not otherwise indicated

Abbreviations: AF = atrial fibrillation, AFl = atrial flutter, SR = sinus rhythm, bpm = beats per minute, ED = emergency department, hsTnT = high-sensitive Troponin-T, ECG = electrocardiography, CVRF = cardiovascular risk factors, BP = blood pressure, CRP = C-reactive protein, n = number

### High-sensitive Troponin-T and atrial fibrillation

When comparing the three hsTnT groups (< 15, 15–50 and > 50 ng/L), there were significant differences in age (*p* < 0.001), duration of AF (*p* = 0.006), symptoms such as palpitations (*p* < 0.001), dyspnoea (*p* = 0.004), chest pain (*p* = 0.027), haemoglobin (*p* < 0.001), CRP (*p* < 0.001) and creatinine (*p* < 0.001) (Table 1).

A total of 285 AF patients were admitted to hospital. There was a statistically significant difference in hsTnT levels in patients admitted to hospital when comparing 2018 with 2020 (*p* = 0.042) (Supplementary Table 1S). As age increased, hsTnT levels also showed a significant rise (*p* < 0.001, correlation coefficient (r) 0.481) (Supplementary Fig. S1).

An association was found between increased creatinine > 100 µmol/L and hsTnT level (*p* < 0.001). Patients with increased creatinine (> 100 µmol/L) had a median hsTnT of 25 (Q1-Q3 14–40) ng/L while those with normal creatinine (< 100 µmol/L) had a hsTnT of 12 (8-21) ng/L. There was no significant association between hsTnT level and other comorbidities. A significant association was found between palpitations and hsTnT level where patients with palpitations had a hsTnT level of 11 (Q1-Q3 7–19) ng/L compared to 21 (11–34) ng/L in patients without (*p* < 0.001). Dyspnoea was also associated with increased hsTnT level where patients with dyspnoea had a median hsTnT of 17 (Q1-Q3 10–29) ng/L compared to 13 (8–23) ng/L in patients without (*p* < 0.001) (Table 2).

**Table 2:** Association between symptoms/comorbidities and high-sensitive Troponin-T (hsTnT) in 625 patients with atrial fibrillation/flutter (AF) at the emergency department (ED) sampled for hsTnT, Vrinnevi Hospital, Norrkoping, Sweden, during years 2018 and 2020

Symptom	Patients with symptom, *n* (%)	Patients without symptom, *n* (%)	hsTnT level, patients with symptom, median (Q1-Q3)	hsTnT level, patients without symptom, median (Q1-Q3)	*p*-value
Palpitations	360 (58)	265 (42)	11 (7–19)	21 (11–34)	< 0.001
Dyspnoea	230 (37)	395 (63)	17 (10–29)	13 (8–23)	< 0.001
Chest pain	135 (22)	490 (78)	18 (10–24)	13 (8–25)	0.191
Fatigue	143 (23)	482 (77)	15 (8–28)	14 (9–24)	0.387
Dizziness	132 (21)	493 (79)	14 (8–23)	14 (9–25)	0.376
Syncope	20 (3)	605 (97)	12 (8–23)	14 (9–25)	0.630
**Comorbidity and CVRF**	**Patients with comorbidity**, n (%)	**Patients without comorbidity**, n (%)	**hsTnT level**,** patients with comorbidity**, **median (Q1-Q3)**	**hsTnT level**,** patients without comorbidity**, **median (Q1-Q3)**	**p-value**
Heart failure	163 (26)	462 (74)	14 (8–24)	14 (9–26)	0.783
Coronary artery disease	107 (17)	518 (83)	15 (9–28)	14 (8–24)	0.400
Type-2 diabetes mellitus	116 (19)	509 (81)	15 (10–22)	14 (8–26)	0.639
Hypertension	440 (70)	185 (30)	13 (8–24)	16 (9–31)	0.080
Creatinine > 100 µmol/L	156 (25)	468 (75)	25 (14–40)	12 (8–21)	< 0.001

Data are presented as number (%) or median (Q1-Q3) if not otherwise indicated

Abbreviations: hsTnT = High-sensitive Troponin-T, AF = atrial fibrillation/flutter, ED = emergency department, CVRF = cardiovascular risk factors, n = number

### Mortality

Among the 625 patients, all-cause mortality was 2% (*n* = 14) at 30 days, and 8% (*n* = 50) at 1 year. The 30-day mortality rates for the hsTnT groups were: 0.3% for hsTnT < 15 ng/L; 3% for hsTnT 15–50 ng/L; and 10% for hsTnT > 50 ng/L (*p* < 0.001). Additionally, the mortality rates for 1-year mortality were: 2% for hsTnT < 15 ng/L; 12% for hsTnT 15–50 ng/L; and 28% for hsTnT > 50 ng/L (*p* < 0.001).

The HR for 30-day mortality, following adjustment for age, tended to be higher in patients with elevated hsTnT and was 4.17 (95% CI 0.49–35.79, *p* = 0.192) for hsTnT levels between 15 and 50 ng/L) and 9.64 (95% CI 0.98–95.30, *p* = 0.053) for patients with hsTnT levels > 50 ng/L in comparison to patients with hsTnT levels < 15 ng/L (Table 3).

**Fig. 2:**
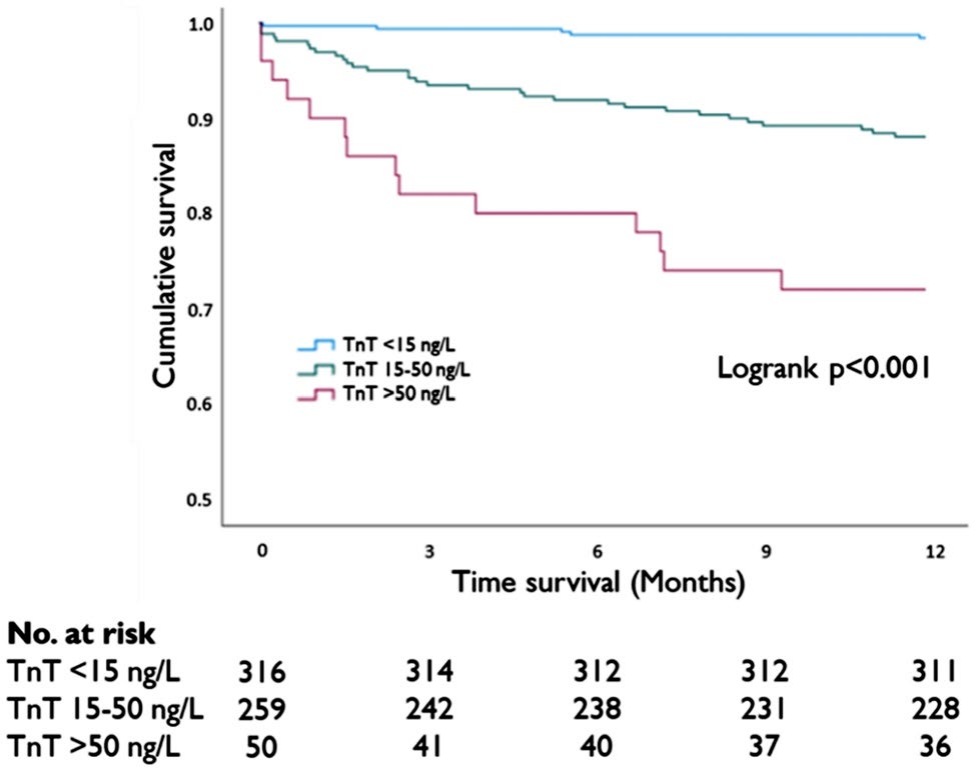
Kaplan-Meier curves and number at risk illustrating 1-year mortality for different Troponin-T levels. TnT = High-sensitive Troponin-T

The HR for 1-year mortality following adjustment for age, was significantly higher in patients with hsTnT levels 15–50 ng/L (HR 4.82, 95% CI 1.81–12.82, *p* = 0.002) and hsTnT levels > 50 ng/L (HR 9.70, 95% CI 3.27–28.74, *p* < 0.001) in comparison to patients with hsTnT levels < 15 ng/L (Table 3). There were also significant differences in 1-year mortality across hsTnT groups, illustrated in the Kaplan-Meier curve (Fig. 2).

**Table 3:** 30-day and 1-year mortality with hazard ratio (HR) and 95% confidence interval (CI) for high-sensitive Troponin-T (hsTnT) groups in 625 patients with atrial fibrillation/flutter (AF) at the emergency department (ED) sampled for Troponin-T, Vrinnevi Hospital, Norrkoping, Sweden, during years 2018 and 2020

hsTnT (ng/L), *n*	30-day mortality, %	Prediction model for 30-day mortality ^a^, HR (95% CI)	*p*-value ^a^
< 15 *n* = 316	0.3	Reference group ^**b**^	-
15–50 *n* = 259	3	4.17 (0.49–35.79)	0.192
> 50 *n* = 50	10	9.64 (0.98–95.30)	< 0.053
**hsTnT (ng/L)**,** n**	**1-year mortality**,** %**	**Prediction model for 1-year mortality**^**a**^, **HR (95% CI)**	**p-value** ^**a**^
< 15 *n* = 316	2	Reference group ^**b**^	-
15–50 *n* = 259	12	4.82 (1.81–12.82)	0.002
> 50 *n* = 50	28	9.70 (3.27–28.74)	< 0.001

Data are presented as number (%) or HR (95% CI) if not otherwise indicated

Abbreviations: HR = hazard ratio, CI = confidence interval, hsTnT = High-sensitive Troponin-T, AF = atrial fibrillation/flutter, ED = emergency department, n = number

^a^ Adjusted for age

^b^ Reference group chosen as most common hsTnT group

Dyspnea was associated with increased mortality (HR 2.45, 95% CI 1.41–4.32, *p* = 0.002), while palpitations were associated with a 76% lower 1-year mortality (HR 0.24, 95% CI 0.13–0.46, *p* < 0.001). Other symptom such as chest pain (HR 1.14, 95% CI 0.59–2.17, *p* = 0.700), fatigue (HR 0.96, 95% CI 0.49–1.87, *p* = 0.893), dizziness (HR 0.71, 95% CI 0.33–1.51, *p* = 0.370), and syncope (HR 0.61, 95% CI 0.09–4.43, *p* = 0.627) were not significantly associated with 1-year mortality (Table 4).

A creatinine over 100 µmol/L was significantly associated with increased 1-year mortality (HR 2.09, 95% CI 1.19–3.69, *p* = 0.010). However, significance disappeared after adjusting for age (HR 1.54, 95% CI 0.86–2.74, *p* = 0.144). No other comorbidity was significantly associated with 1-year mortality: HF (HR 1.47, 95% CI 0.82–2.65, *p* = 0.193), CAD (HR 1.06, 95% CI 0.52–2.19, *p* = 0.868), T2DM (HR 1.38, 95% CI 0.72–2.63, *p* = 0.337), and HT (HR 0.62, 95% CI 0.35–1.09, *p* = 0.094) (Table 4).

**Table 4:** 1-year mortality with hazard ratio (HR) and 95% confidence interval (CI) for symptoms and comorbidities in 625 patients with atrial fibrillation/flutter (AF) at the emergency department (ED) sampled for high-sensitive Troponin-T (hsTnT), Vrinnevi Hospital, Norrkoping, Sweden, during years 2018 and 2020

Symptom	1-year mortality, *n* (%)	Prediction model for 1-year mortality, HR (95% CI)	*p*-value	Prediction model for 1-year mortality ^a^, HR (95% CI)	*p*-value ^a^
Palpitations	13 (4)	0.24 (0.13–0.46)	< 0.001	0.33 (0.18–0.63)	< 0.001
Dyspnoea	29 (13)	2.45 (1.41–4.32)	0.002	2.18 (1.24–3.83)	0.007
Chest pain	12 (9)	1.14 (0.59–2.17)	0.700	0.94 (0.49–1.81)	0.855
Fatigue	11 (8)	0.96 (0.49–1.87)	0.893	0.97 (0.50–1.90)	0.937
Dizziness	8 (6)	0.71 (0.33–1.51)	0.370	0.79 (0.37–1.68)	0.534
Syncope	1 (5)	0.61 (0.09–4.43)	0.627	0.69 (0.10–5.03)	0.718
**Comorbidity and CVRF**	**1-year mortality**, n (%)	**Prediction model for 1-year mortality**, HR (95% CI)	**p-value**	**Prediction model for 1-year mortality**^**a**^, HR (95% CI)	p-value ^a^
Heart failure	17 (10)	1.47 (0.82–2.65)	0.193	1.56 (0.87–2.80)	0.136
Coronary artery disease	9 (8)	1.06 (0.52–2.19)	0.868	0.94 (0.45–1.94)	0.862
Type-2 diabetes	12 (10)	1.38 (0.72–2.63)	0.337	1.46 (0.76–2.79)	0.258
Hypertension	30 (7)	0.62 (0.35–1.09)	0.094	0.63 (0.36–1.11)	0.110
Creatinine > 100 µmol/L	20 (13)	2.09 (1.19–3.69)	0.010	1.54 (0.86–2.74)	0.144

Abbreviations: HR = hazard ratio, CI = confidence interval, AF = atrial fibrillation/flutter, ED = emergency department, hsTnT = high-sensitive Troponin-T, CVRF = cardiovascular risk factors, n = number

^a^ Adjusted for age

## Discussion

There have been few previous studies assessing the relationship between levels of hsTnT and mortality of patients attending the ED with a primary diagnosis of AF. Other studies analysing the relationship between hsTnT and mortality have used different settings, either with different cut-off values for hsTnT or not only including patients at the ED with primary AF diagnosis [13–15]. Considering this, the results of this study might be of particular interest for practicing physicians at the emergency department for guidance in treatment of AF patients with specific hsTnT levels.

Patients attending the ED and who were admitted to the hospital had higher hsTnT levels than those discharged from the ED. Other studies have shown an increase in adverse outcomes such as stroke, major bleeding, and mortality in patients with AF and elevated hsTnT [8, 9]. This may explain why higher hsTnT levels are associated with hospital admission since these patients usually have a greater disease burden. Elevated hsTnT also resulted in serial hsTnT sampling in patients with low risk of CAD, which may partly be explained by routines at the hospital where multiple sampling is recommended in patients with elevated first hsTnT.

Levels of hsTnT were significantly higher in 2020 than 2018. One contributing factor to this might be the Covid-19 pandemic which quickly spread during 2020. As a result of this, patients with lighter symptoms and medical burden might choose to not attend the ED, increasing the ratio of patients with higher cardiovascular burden, and thus higher hsTnT.

hsTnT were in three categories (< 15, 15–50 and > 50 ng/L), which can be considered as normal, minor hsTnT elevation, and elevated hsTnT. Physicians may consider hsTnT 15–50 ng/L, and absence of chest pain, as a clinical grey zone, and the aim was therefore to analyse whether patients with hsTnT 15–50 ng/L had an increased risk of mortality since this may be of particular interest for practicing physicians. Also, the hsTnT 15–50 ng/L cut-off have previously been shown by other authors, to be of clinical significance with increased mortality [9].

We found a significant correlation between age and hsTnT level; hsTnT increasing with age. A similar association between high age and elevated hsTnT levels has also been reported in other studies [11]. Increased mortality in patients with AF and mildly elevated hsTnT levels may partly be explained by the higher incidence of coronary artery disease in older patients [16]. The prevalence of CAD in this study was 17%, compared to the prevalence in Sweden of 7% in the age group 65–75 years of age and 30% in patients 70–79 [17, 18]. There was no significant association between CAD and 1-year mortality in our cohort. 97% of all AF patients with chest pain as presenting symptom at the ER were sampled for hsTnT.

No comorbidity covered in this study appeared to have a significant impact on 1-year mortality. This may be a result of various reasons such as well optimized treatment of comorbidities since they were diagnosed or a short follow-up period of 12 months in this study. Increased creatinine correlated with increased hsTnT levels, which has previously been noted and may depend on an association between kidney diseases and various comorbid cardiovascular diseases and impaired renal filter function, which leads to elevated hsTnT levels [19].

Heart failure (HF) was seen in 26% of the cohort and in 25% of the eldest age group, which is much higher than the 10% prevalence in the elderly Swedish population [17, 18]. However, other studies on AF patients have reported 24–37% prevalence, so the figures in the present study seem adequate and we know that HF is associated with atrial fibrillation [20, 21]. The coexistence of AF and HF may also elevate the likelihood of an acute event causing the patient to seek acute medical care.

Interestingly, palpitations were associated with lower hsTnT levels and lower 1-year mortality. It could be that palpitations represent isolated new onset AF with a more favourable outcome than AF with the other symptoms studied that usually indicate more severe heart disease. Furthermore, the fact that palpitations were highly associated with lower hsTnT levels could explain the lower mortality in this group compared, for example, to dyspnoea with significantly increased hsTnT levels and 1-year mortality.

### Strengths and limitations

A limitation of this study is that no differentiation was made between diagnoses with ICD-10 code I48, in particular between paroxysmal and persistent AF. The initial diagnosis came from the emergency department, but for those admitted to the ward, it might have been updated by the time they were discharged. Despite this being a single-centre study, with inherent limitations, our ED has a large catchment area of 170,000 inhabitants which enabled us to analyse a complete real-life cohort of all AF patients attending the ED in this region. A larger prospective study would be needed to have the power to compare 30-day mortality with elevated hsTnT.

It is also worth mentioning that the hospital ED uses RETTS triage system, which gives physicians recommended blood test samplings depending on patients’ main symptoms. In patients with palpitations or abnormal heart rhythm, hsTnT is recommended. As a result of this, a large percentage of patients with atrial fibrillation have been sampled with hsTnT. However, it is important to note that 146 patients were excluded due to not being sampled with hsTnT. This could be due to the physician’s clinical assessment in the ER indicating a low risk of ischemic heart disease, leading to the decision not to test with hsTnT, or it might have been overlooked.

## Conclusion

Higher hsTnT increased hazard ratios for 30-day and 1-year mortality in patients attending the ED with AF. Elevated hsTnT level independently of age appeared to be a risk factor for 1-year mortality. We stress the association between AF together with dyspnoea and increased mortality. This may alert physicians to lower their threshold for hospital admission in this group of patients.

## Electronic supplementary material

Below is the link to the electronic supplementary material.

Supplementary Material 1

## Data Availability

The datasets generated and/or analysed in the present study are not publicly available according to Swedish laws and regulations but are available from the corresponding author upon reasonable request.
