# Supplementary material for: Troponin-T as predictor of mortality in patients attending the emergency department with atrial fibrillation
Source: BMC Cardiovasc Disord. 2024 Dec 20;24:719. doi: 10.1186/s12872-024-04388-8 (PMC11660876; doi:10.1186/s12872-024-04388-8)
Supplement: Supplementary file 1 — Supplementary Material 1 [file 12872_2024_4388_MOESM1_ESM.docx]

| **Table 1S.** **Number and characteristics of 625 patients** with Atrial fibrillation/flutter (AF) at the Emergency department (ED) sampled for Troponin-T, Vrinnevi Hospital, Norrkoping, Sweden, during years 2018 and 2020. | | | | |
| --- | --- | --- | --- | --- |
| **Characteristic** | **2018** | **2020** | **All** | **p-value** |
| Patients included in the study, n (%) | 305 (57) | 320 (65) | 625 (61) | <0.001 |
| **Discharged,** n (%) | 158 (52) | 182 (57) | 340 (54) | 0.203 |
| TnT level ng/L, median (Q1-Q3 ~~IQR~~) | 10 (7-19 ~~12~~) | 12 (8-18 ~~10~~) | 11 (8-19 ~~11~~) | 0.129 |
| **Admitted to hospital,** n (%) | 147 (42) | 138 (43) | 285 (46) | 0.203 |
| TnT level ng/L, median (Q1-Q3 ~~IQR~~) | 18 (10-29 ~~19~~) | 21 (12-39 ~~27~~) | 20 (11-36 ~~25~~) | 0.042 |
| Multiple sampling, n (%) | 92 (63) | 99 (72) | 189 (66) | 0.061 |
| Data are presented as number (%) or median (Q1-Q3) if not otherwise indicated.  Abbreviations: AF = atrial fibrillation/flutter, ED = emergency department, TnT = Troponin-T, n = number | | | | |
